# Physical activity measures in patients with myalgic encephalomyelitis/chronic fatigue syndrome: correlations between peak oxygen consumption, the physical functioning scale of the SF-36 questionnaire, and the number of steps from an activity meter

**DOI:** 10.1186/s12967-020-02397-7

**Published:** 2020-06-08

**Authors:** C. M. C. van Campen, Peter C. Rowe, Freek W. A. Verheugt, Frans C. Visser

**Affiliations:** 1 Stichting CardioZorg, Planetenweg 5, 2132 HN Hoofddorp, The Netherlands; 2 Department of Pediatrics, Johns Hopkins University School of Medicine, Baltimore, USA; 3 Onze Lieve Vrouwe Gasthuis (OLVG), Oosterpark 9, 1091 AC Amsterdam, The Netherlands

**Keywords:** Sensewear™ armband, Chronic fatigue syndrome, Spiro-ergometry, Peak VO_2_, SF 36 questionnaire, Myalgic encephalitis

## Abstract

**Background:**

Most studies to assess effort intolerance in patients with myalgic encephalomyelitis/chronic fatigue syndrome (ME/CFS) have used questionnaires. Few studies have compared questionnaires with objective measures like an actometer or an exercise test. This study compared three measures of physical activity in ME/CFS patients: the physical functioning scale (PFS) of the SF-36, the number of steps/day (Steps) using an actometer, and the %peak VO_2_ of a cardiopulmonary stress test.

**Methods:**

Female ME/CFS patients were selected from a clinical database if the three types of measurements were available, and the interval between measurements was ≤ 3 months. Data from the three measures were compared by linear regression.

**Results:**

In 99 female patients the three different measures were linearly, significantly, and positively correlated (PFS vs Steps, PFS vs %peak VO_2_ and Steps vs %peak VO_2_: all P < 0.001). Subgroup analysis showed that the relations between the three measures were not different in patients with versus without fibromyalgia and with versus without a maximal exercise effort (RER ≥ 1.1). In 20 patients re-evaluated for symptom worsening, the mean of all three measures was significantly lower (P < 0.0001), strengthening the observation of the relations between them. Despite the close correlation, we observed a large variation between the three measures in individual patients.

**Conclusions:**

Given the large variation in ME/CFS patients, the use of only one type of measurement is inadequate. Integrating the three modalities may be useful for patient care by detecting overt discrepancies in activity and may inform studies that compare methods of improving exercise capacity.

## Background

Myalgic Encephalomyelitis/Chronic Fatigue Syndrome (ME/CFS) is a serious and chronic disease marked by impairment of the activities the affected individual previously tolerated [1–3]. One of the defining characteristics of ME/CFS is effort intolerance and a prolonged recovery after increased activity [4, 5]. The pathophysiology of the exercise intolerance is not known but involves both metabolic abnormalities of skeletal muscles as well as central nervous system abnormalities [5–11]. By definition, ME/CFS involves some degree of disability, defined as “any restriction or lack (resulting from an impairment) of ability to perform an activity in the manner or within the range considered normal for a human being” [12].

Disability in ME/CFS is multi-factorial, with social, physical, mental, training and labour dimensions. The majority of ME/CFS studies have employed questionnaires to describe the extent of disability. One of the commonly used questionnaires is the SF-36 and especially the physical functioning scale of this questionnaire [13–20].

Objective measures of functional status and wellbeing have the potential to augment and improve the validity of questionnaire measures. Exercise intolerance can be quantified by using an activity meter [21] and by measuring peak oxygen consumption on a cardiopulmonary stress test (CPET) [22–33]. We are not aware of studies measuring how well questionnaires and these two objective measurements correlate in ME/CFS populations. The aim of this study was to correlate peak oxygen consumption with the number of steps on the Sensewear™ armband and with the physical functioning scale of the SF-36 in ME/CFS patients. We elected to answer this question by studying female ME/CFS patients because of differences in peak oxygen consumption between males and females, and possible gender differences in the clinical phenotype of the disease [34–36].

## Methods

### Participants

This was a retrospective study of patients referred between 2012 and 2019 to the Stichting CardioZorg, a cardiology clinic that specializes in the assessment and treatment of those with CFS and ME. All eligible participants had been referred by their general practitioners for the diagnosis of ME/CFS. Patients underwent a detailed clinical history, physical examination, laboratory analysis, ECG and echocardiography. Based on their symptoms the diagnosis of chronic fatigue syndrome (CFS) according to the Fukuda Criteria [1] and myalgic encephalomyelitis (ME) according to the international ME criteria [2] was established. In all patients alternative diagnoses which could explain the fatigue and other symptoms were ruled out. We excluded patients with a body mass index of ≥ 50 because the normal reference values for the CPET [37] were based on female healthy volunteers with a BMI up to 50. The diagnosis of fibromyalgia was based on either the previous diagnosis of a rheumatologist or based on the American College of Rheumatology questionnaire 2010 [38]. Those with fibromyalgia did not have arthritic or secondary forms of the disorder.

A subset of patients completed the SF-36 questionnaire, wore a Sensewear™ armband for 5 days and underwent cardiopulmonary exercise testing. These tests were primarily performed to demonstrate the degree of disability because of conflicts with the social security administration.

From among those who had undergone all three tests, to minimize between-measures variability in functional status, we selected for study those in whom the interval between the SF-36 questionnaire, the Sensewear™ measurements, and the cardiopulmonary stress test was less than 3 months. In addition, patients who were re-evaluated because of worsening of symptoms were analyzed separately.

The study was carried out in accordance with the Declaration of Helsinki. The use of clinical data for descriptive studies was approved by the ethics committee of the Slotervaart Hospital, the Netherlands (P1450). All patients give informed consent to analyze their data.

### Study measures

Cardiopulmonary exercise testing (CPET): patients underwent a symptom-limited exercise test on a cycle ergometer (Excalibur, Lode, Groningen, The Netherlands) according to a previously described protocol [22]. A RAMP workload protocol was used varying between 10 and 30 W/min increases, depending on sex, age and expected exercise intolerance. Oxygen consumption (VO_2_), carbon dioxide release (VCO_2_) and oxygen saturation were continuously measured (Cortex, Procare, The Netherlands), and displayed on screen using Metasoft software (Cortex, Biophysic Gmbh, Germany). An ECG was continuously recorded and blood pressures were measured continuously using the Nexfin device (BMEYE, Amsterdam, The Netherlands) [39]. The test was supervised by an experienced cardiologist. Patients were encouraged by standard phrases each minute to perform maximally to the point of exhaustion. The mean of the VO_2_ measurements of the last 15 s before ending the exercise (peak VO_2_) was taken, and expressed as a percentage of the normal values of a population study: %peak VO_2_ [37]. We assessed the mean respiratory exchange ratio (RER; VCO_2_/VO_2_) of the last 15 s to determine the influence of this measure of maximal effort on the results. Immediately after the test the attending cardiologist noted the primary reason for termination of the exercise and judged whether motivation and efforts during exercise were optimal for the individual patient.

Sensewear™ armband (BodyMedia, Pittsburgh, PA, USA) measurements: Patients wore the armband for approximately 4 days and were advised to remove the armband only during showering or bathing. From the armband data, the number of steps was recorded and normalized to 24 h. The coefficient of variation of the number of steps per day was calculated. After wearing the armband patients were asked if the 4 days were “average” days. If there was a gross over- or under-activity, patients were excluded from the analysis. Furthermore, patients who wore the armband less than 23 h per day were excluded.

SF-36 physical functioning subscale: this subscale of the SF-36 asks whether the respondent’s health limits activities performed during a typical day a lot, a little, or not at all. The Dutch version of the SF-36 for physical functioning [40] was used. The scores of the 10 items of the questionnaire regarding physical activity were transformed into a scale ranging from 0 to 100%.

### Data analysis

Data were analyzed using the statistical package of Graphpad Prism version 6.05 (Graphpad software, La Jolla, California, USA). All continuous data were tested for normal distribution using the D’Agostino–Pearson omnibus normality test, and presented as mean (SD) or as median with the IQR, where appropriate.

Linear regression was performed to assess the relation between measurements (physical function subscale of SF-36, number of steps, and CPET percentage peak VO_2_).

A paired T test was performed on patients who underwent re-evaluation because of worsening symptoms. Because of the multiple comparisons a more conservative P value was chosen in which a P < 0.01 was considered significantly different.

## Results

Between October 2012 and January 2019, 844 patients visited the outpatient clinic of the Stichting CardioZorg and were diagnosed ME/CFS. In 197 female patients all three studies were available. Of these, 110 had all three studies performed within 3 months. Of the 110, 4 patients were excluded because of gross under- or over-activity compared to their average daily activity, 1 because of an insufficient armband wear time, and 1 because of recording artifacts. Two patients were excluded because of motion artifacts (both had a ride of more than 1 h on a motorbike/scooter, during which the Sensewear™ device reflected vigorous activity). Three patients experienced an allergic skin reaction to the armband that disrupted the recording. Thus, 99 patients were included in the analysis. All electrocardiograms were normal; the echocardiograms were either normal or showed minor abnormalities like mild valvular regurgitation and mild left atrial enlargement. No left ventricular dysfunction was present. No patients had either diabetes mellitus or moderate to severe COPD. Sixteen patients (all with co-morbid fibromyalgia) were taking prescribed analgesic medications at the time of the study (consisting of 2 taking gabapentin, 2 duloxetine, 3 pregabalin, 2 oxycodone, 3 naltrexone in low doses, 2 tramadol, and 2 amitriptyline). Four were taking benzodiazepine medications, primarily for sleep (3 oxazepam, 1 temazepam). Two intermittently took non-steroidal anti-inflammatory medications (1 diclofenac, 1 ibuprofen).

Nineteen patients were re-evaluated after an interval of at least a year because of symptom worsening and underwent repeat CPET, activity tracking, and completion of the SF-36 questionnaire to determine whether these measures changed in association with the change in clinical status.

Table 1 shows the characteristics of the 99 female study participants with ME/CFS. The interval between the CPET and SF questionnaire was less than 1 week in all participants. The median interval between the SF-36 questionnaire and Sensewear™ measurements was 2 months (IRQ 1–3 months) and the median interval between the CPET and Sensewear™ was 1 month (IRQ: 1–2 months). We confirmed by chart review that no patient experienced a substantial change in function in the interval between measurements. Of the Sensewear™ measurements the between-days variation of the number of steps of an individual patient was expressed as the coefficient of variation. The median coefficient of variation of the number of steps in the total population was 7% (IQR: 3–16%). CPET data: using a RER ≥ 1.1 as a criterion for a maximal exercise (41), 53 patients (54%) reached a RER above 1.1. The primary reason for termination of the exercise was muscle strength exhaustion or muscle pain in 86 patients (87%), shortness of breath in 9 patients (9%) and other reasons in 4 patients (4%). The attendant cardiologist judged patient motivation and efforts to be maximal in all patients.

**Table 1:** Demographic features and main activity measures

Number of patients	99
Age in years	41 (11)
Fibromyalgia (number/percentage)	58 (59%)
BMI in kg/m^2^	24.4 (5.2)
Disease duration in years (median/IQR)	12 (6–7)
SBP at rest in mmHg	125 (12)
DBP at rest in mmHg	78 (8)
Heart rate at rest in bpm	80 (13)
Number of steps/day (median/IQR)	4683 (3269–7399)
SF-36 Physical Activity scale	46 (21)
%peak VO_2_	76 (18)
RER	1.11 (0.11)
RER ≥ 1.10 (number/percentage)	53 (54%)

All values are mean (SD) unless otherwise noted

*BMI* body mass index, *no* number, *SBP* systolic blood pressure, *DBP* diastolic blood pressure, *HR* heart rate, *RER* respiratory exchange ratio, *%peak VO*_*2*_ oxygen consumption at peak exercise as percentile of a reference population

**Fig. 1:**
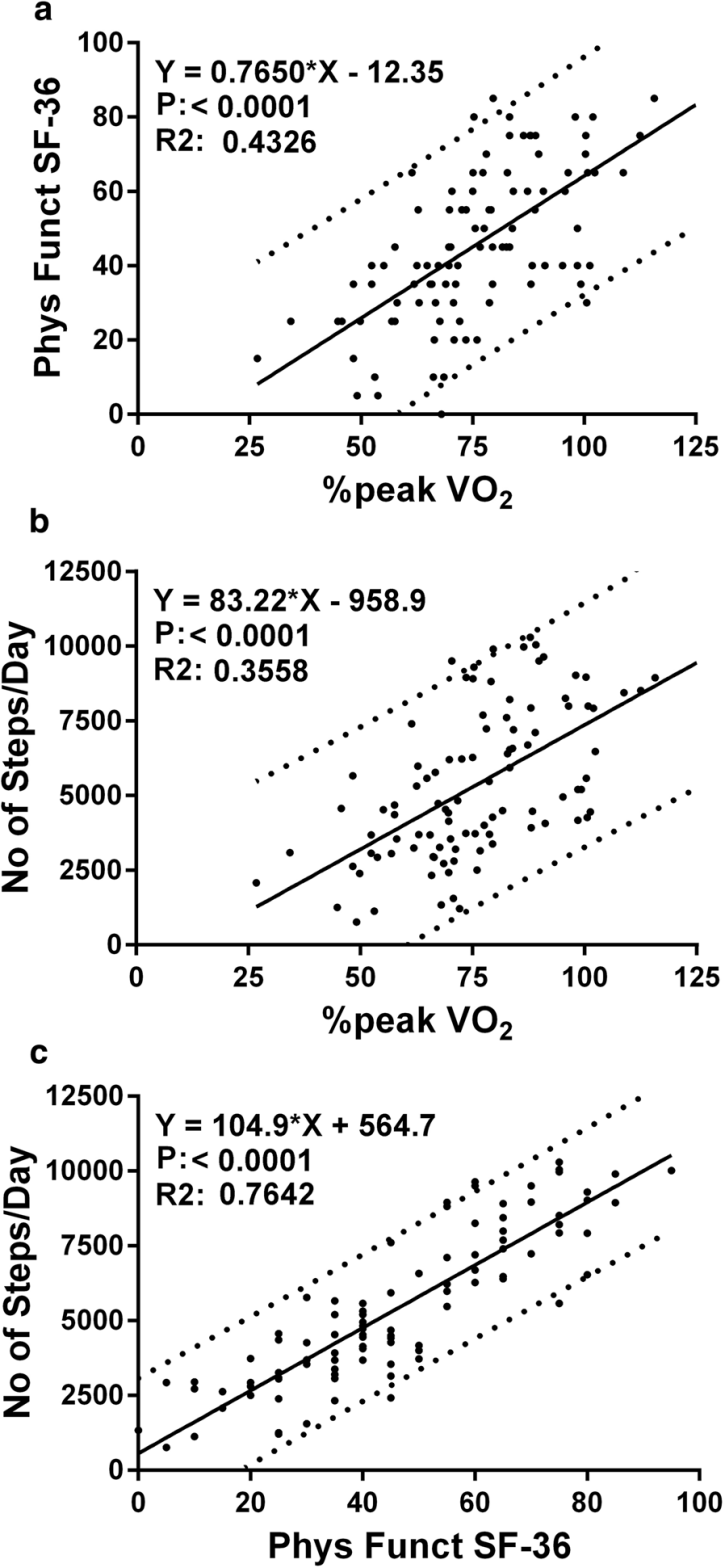
Correlations and 95% prediction intervals between the three measures of physical activity. **a** The correlation between the SF-36 physical functioning scale and the percentage peak VO_2_ relative to controls [37]; **b** The correlation between the number of steps/day and the percentage peak VO_2_; **c** The correlation between the number of steps/day and the SF-36 physical functioning scale. All correlations were highly significant. Phys Funct SF-36: the physical functioning scale of the SF-36 questionnaire; %peak VO_2_ of Norm: peak VO_2_ oxygen uptake relative to controls

Figure 1 shows the correlations and the 95% prediction intervals between the %peak VO_2_, the SF-36 physical functioning scale, and the number of steps per day. All relations were highly significant (all P < 0.0001).

To evaluate whether the association between measures was affected by conventional definitions of effort expended, as measured by the RER, we performed a subgroup analysis in ME/CFS patients with and without a RER ≥ 1.1. To evaluate the influence of muscle pain on exercise termination we also compared ME/CFS patients with and without fibromyalgia. The RER in ME/CFS patients with fibromyalgia (n = 58) was significantly lower (1.07 (0.11)) compared to ME/CFS patients without fibromyalgia (n = 41) (RER 1.17 (0.10)), P < 0.0001.

Table 2 shows that there is no significant difference between patients with a peak RER ≥ 1.1 vs patients with a peak RER < 1.1 in the number of steps/day, the physical functioning scale of the SF-36, or the %peak VO_2_. Also there was no difference in these parameters in patients with and without fibromyalgia as shown in Table 3.

**Table 2:** Subgroup analysis of ME/CFS patients with and without a RER ≥ 1.1

	RER ≥ 1.1	RER < 1.1	P-value
Number/percentage	53 (54%)	46 (46%)	
Number of steps/day (median/IQR)	4683 (3350–7932)	4779 (3352–7279)	0.30
SF-36 Physical activity	49 (20)	38 (22)	0.03
%peak VO_2_	78 (18)	71 (18)	0.09

**Table 3:** Subgroup analysis of ME/CFS patients with and without fibromyalgia

	Fibromyalgia +	Fibromyalgia −	P-value
Number/percentage	58 (58%)	41 (41%)	
Number of steps/day (median/IQR)	4527 (3264–6235)	6401 (3531–8123)	0.14
SF-36 Physical activity	42 (19)	52 (22)	0.02
%peak VO_2_	74 (19)	80 (16)	0.08

All values are mean (SD) unless otherwise noted. For abbreviations see Table 1

**Fig. 2:**
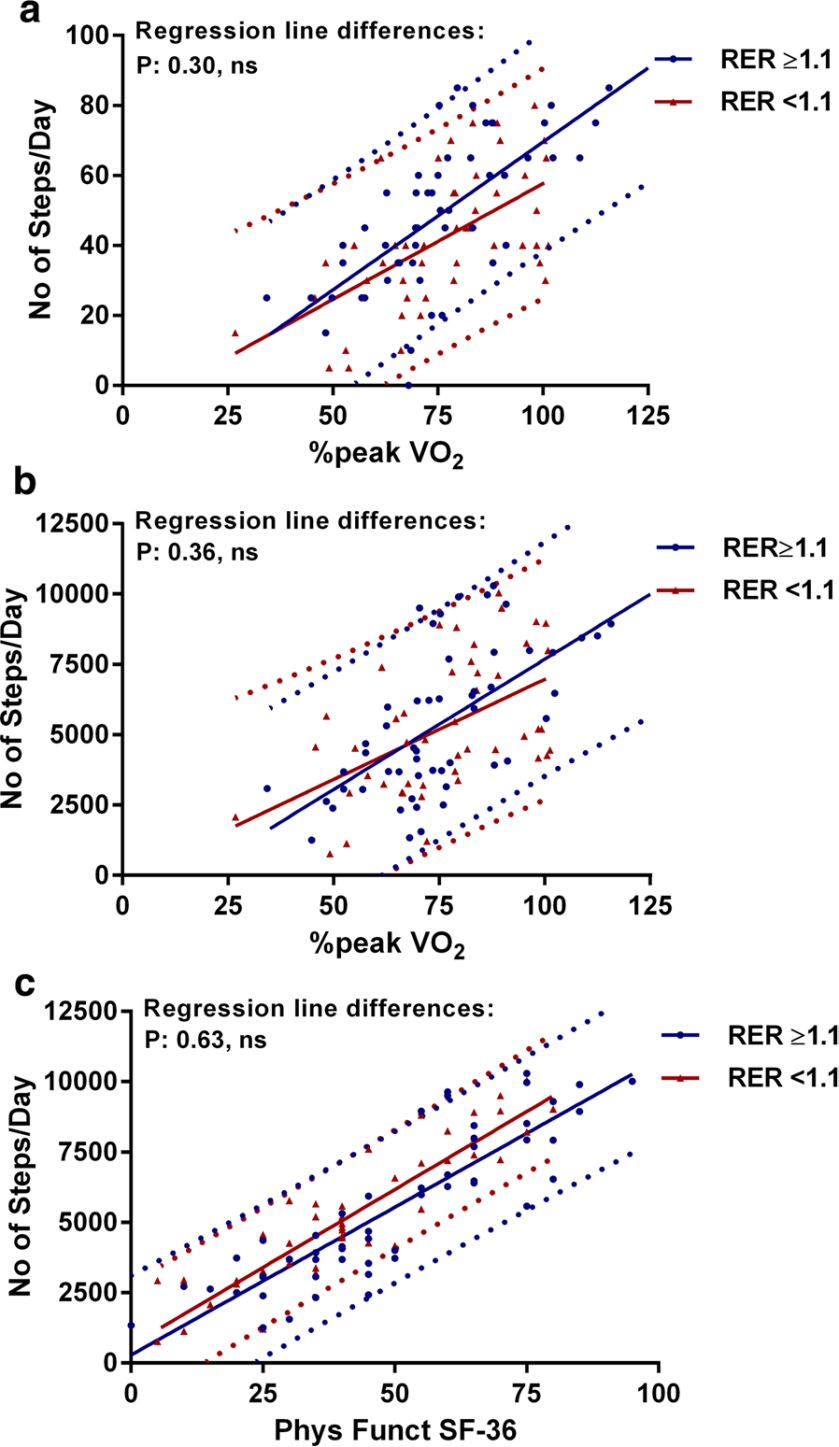
Correlations and 95% prediction intervals between the three measures in those with and without RER ≥ 1.1. **a** The correlation between the SF-36 physical functioning scale and the percentage peak VO_2_, **b** The correlation between the number of steps/day and the percentage peak VO_2_ and **c** shows the correlation between the number of steps/day and the SF-36 physical functioning scale. No significant differences were found in comparing RER < 1.1 and RER ≥ 1.1. Phys Funct SF-36: the physical functioning scale of the SF-36 questionnaire; %peak VO_2_ of Norm: peak VO_2_ oxygen uptake relative to controls

**Fig. 3:**
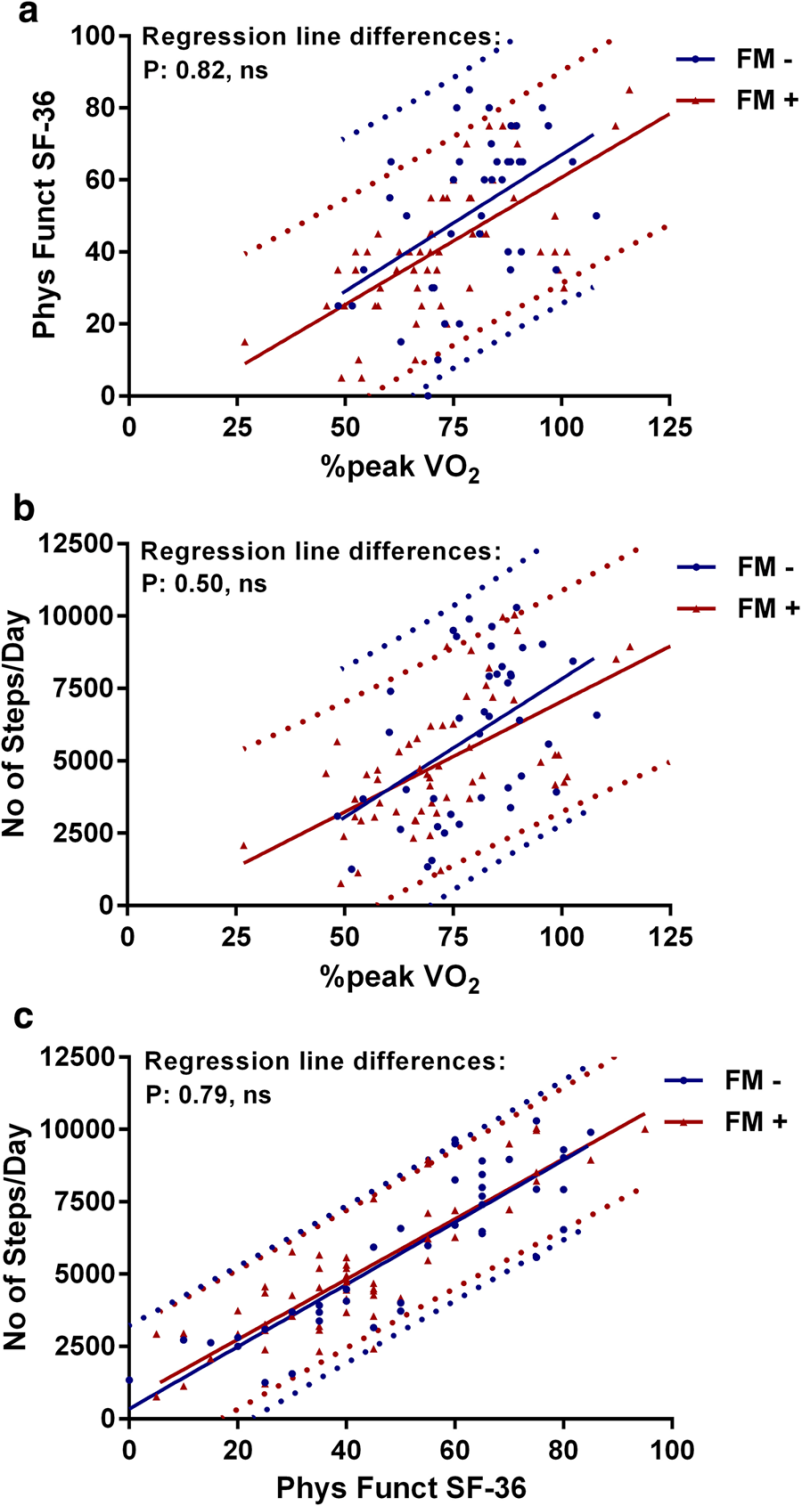
Correlations and 95% prediction intervals between the three measures in those with and without fibromyalgia. **a** The correlation between the SF-36 physical functioning scale and the percentage peak VO_2_, **b** the correlation between the number of steps/day and the percentage peak VO_2_ and **c** the correlation between the number of steps/day and the SF-36 physical functioning scale. No significant differences were found in comparing the absence or presence of fibromyalgia. Phys Funct SF-36: the physical functioning scale of the SF-36 questionnaire; %peak VO_2_ of Norm: peak VO_2_ oxygen uptake relative to controls

Figures 2 and 3 show the correlations and the 95% prediction intervals between the %peak VO_2_, the SF-36 physical functioning scale, and the number of steps per day in patients with and without a RER > 1.1 (Fig. 2) and in patients with and without fibromyalgia (Fig. 3). In patients with a RER ≤ 1.1 the relation between the %peak VO_2_ and the number of steps per day was significant at the level of P < 0.005, all other relations in patients with and without a RER > 1.1 were significant at the level of P < 0.0001. There were no significant differences between the regression lines between patients with and without a RER > 1.1. In patients without fibromyalgia the relation between the %peak VO_2_ and the SF-36 physical functioning scale and between the %peak VO_2_ and the number of steps per day were significant at the level of P < 0.005. All other relations in patients with and without fibromyalgia were significant at the level of P < 0.0001. There were no significant differences between the regression lines in patients with and without fibromyalgia.

**Fig. 4:**
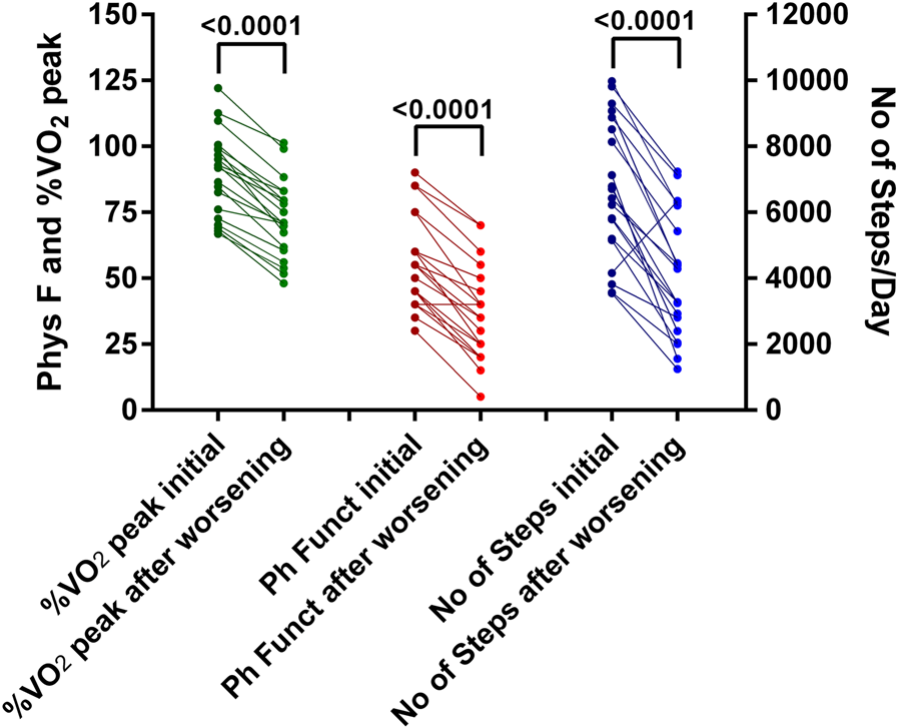
Changes in the three measures in 20 individuals re-tested after experiencing a clinical deterioration. Individual changes in the %peak VO_2_ of normal, the physical functioning scale of the SF-36 questionnaire, and the number of steps/day in 20 patients undergoing re-evaluation because of worsening of symptoms. All mean measurements during the re-evaluation showed a significant deterioration (P < 0.0001) in comparison to the initial evaluation. Phys F: Physical Functioning scale of the SF-36 questionnaire; %peak VO_2_: peak VO_2_ oxygen uptake relative to controls [37]; No of steps: number of steps per day

Figure 4 shows the % of normal peak oxygen consumption, the physical functioning scale of the SF-36, and the number of steps/day in 20 patients undergoing re-evaluation because of worsening of symptoms. Re-evaluation was performed a median of 19 months after the initial visit (IRQ: 14–24 months). All three types of measurements showed a significant deterioration for the number of steps/day, for the physical functioning scale of the SF-36, and for the %peak VO_2_ in comparison to the initial evaluation (all P < 0.0001).

**Fig. 5:**
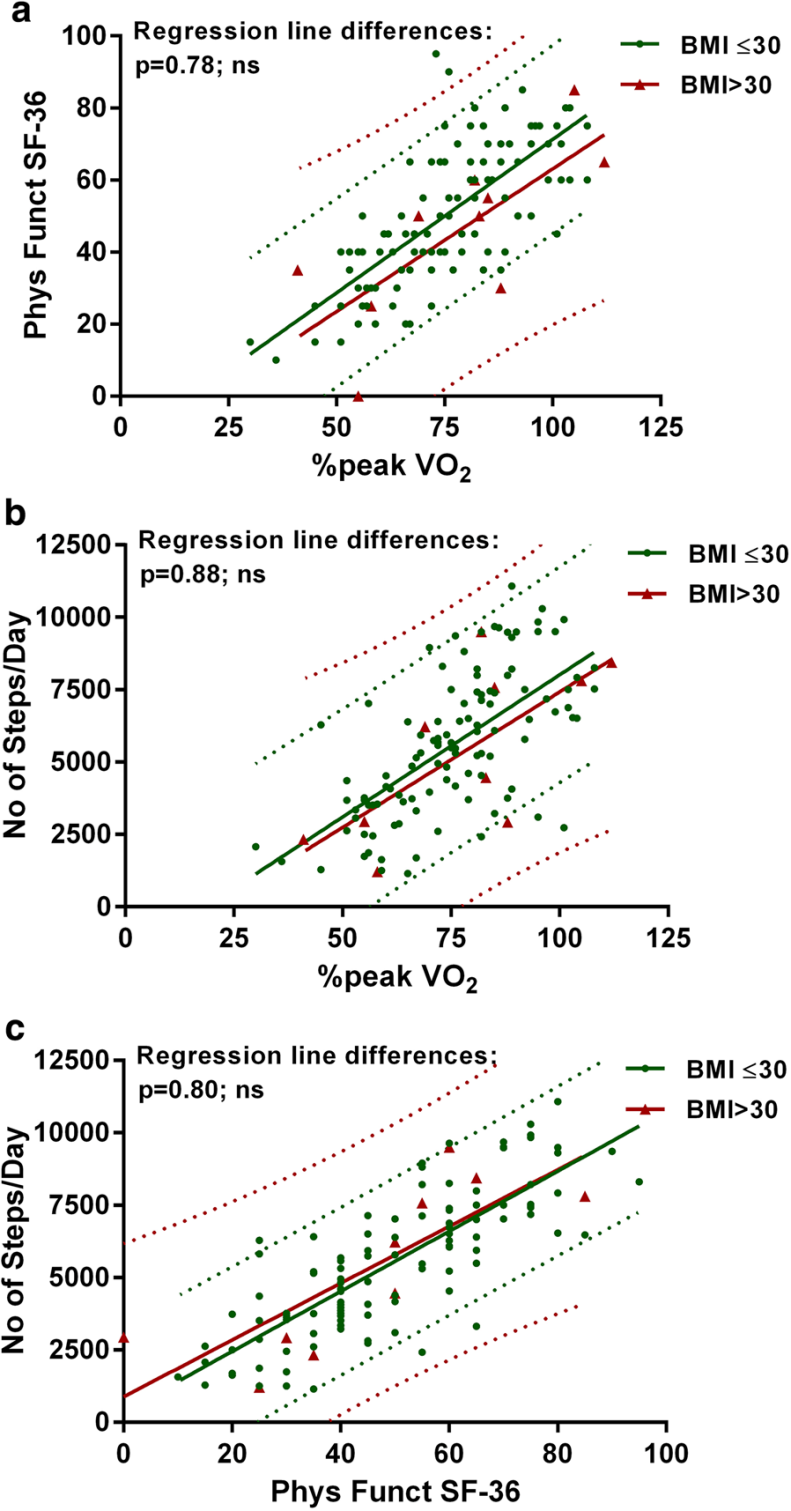
Correlations and 95% prediction intervals between the three measures in those with a BMI ≤ 30 and > 30. **a** The correlation between the SF-36 physical functioning scale and the percentage peak VO_2_, **b** the correlation between the number of steps/day and the percentage peak VO_2_ and **c** the correlation between the number of steps/day and the SF-36 physical functioning scale. No significant differences were found in comparing those with a BMI ≥ 30 or > 30. Phys Funct SF-36: the physical functioning scale of the SF-36 questionnaire; %peak VO_2_ of Norm: peak VO_2_ oxygen uptake relative to controls

**Fig. 6:**
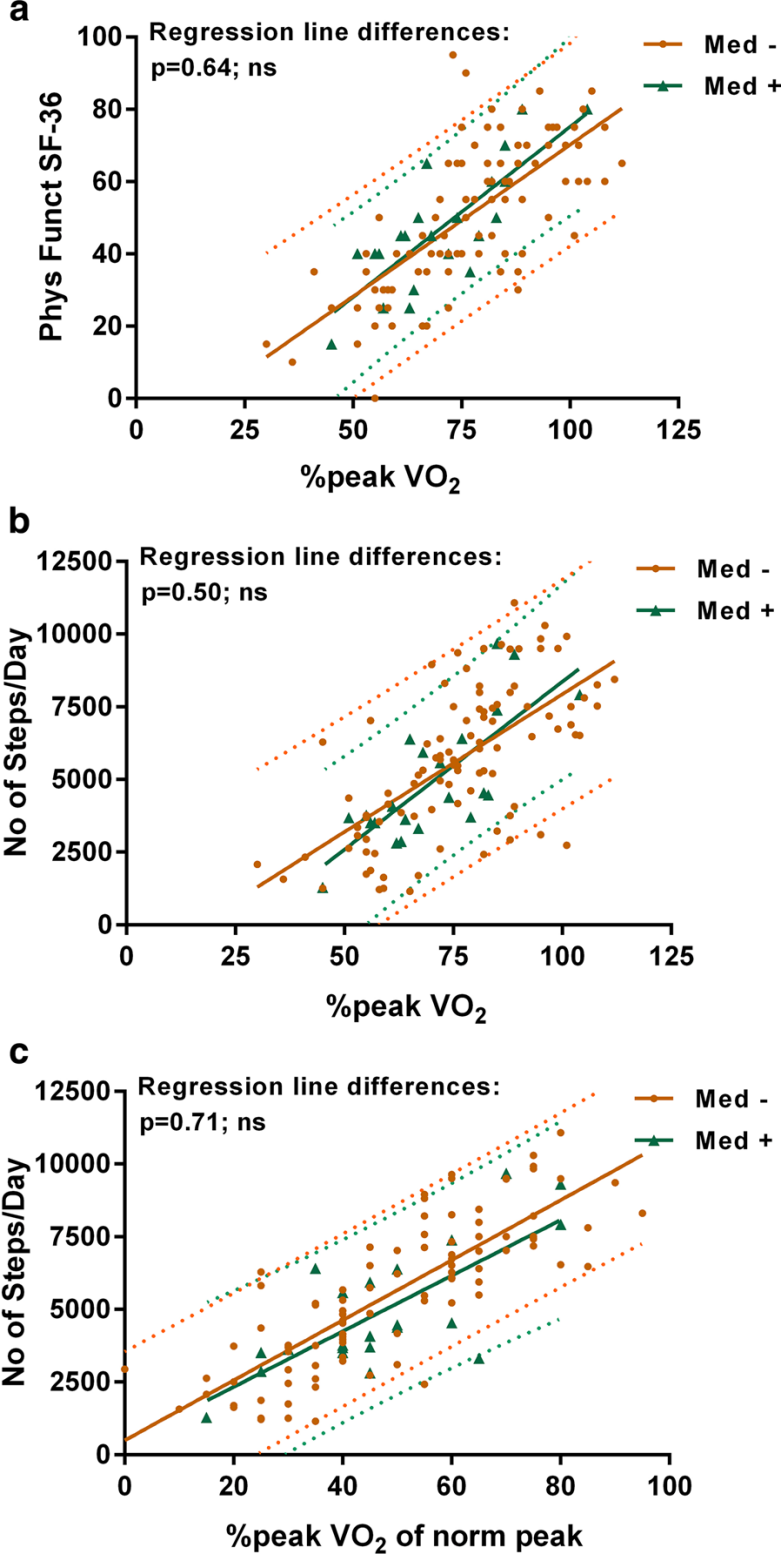
Correlations and 95% prediction intervals between the three measures in those taking medication at the time of the study. **a** The correlation between the SF-36 physical functioning scale and the percentage peak VO_2_, **b** the correlation between the number of steps/day and the percentage peak VO_2_ and **c** the correlation between the number of steps/day and the SF-36 physical functioning scale. No significant differences were found in comparing those being treated with medications or not. Phys Funct SF-36: the physical functioning scale of the SF-36 questionnaire; %peak VO_2_ of Norm: peak VO_2_ oxygen uptake relative to controls

To explore whether obesity influenced the activity outcomes, we compared the physical function measures based on body mass index. Figure 5 shows that the regression lines between %peak VO_2_ and SF-36 score, %peak VO_2_ and steps per day, and SF-36 and steps per day did not differ between those with a BMI < 30 versus > 30 (P values 0.78–0.88). To evaluate whether being on medications at the time of the test influenced the findings, we also compared the groups who were taking medications with those who were on no medications. Figure 6 shows no difference in the three physical function measures based on medication status (P values 0.50–0.71).

## Discussion

The main finding of this study was that all disability measurements were highly significantly correlated: the physical functioning scale of the SF-36, the number of steps on an activity meter, and the percentage peak VO_2_ relative to a reference value of healthy controls. Few studies have been performed to validate disability measurements in ME/CFS patients, especially with the use of more objective measures other than history taking. To the best of our knowledge this is the first study to have measured all three in determining activity levels in ME/CFS patients.

In comparison to values reported in other ME/CFS populations, our study participants had similar steps per day [21], and %peak VO_2_ values [22, 27, 30]. The reported median or mean scores for the physical functioning scale of the SF-36 questionnaire vary widely between studies ranging between 17 and 59% [13–18]. This large variation is probably due to patient selection. Inclusion of more severely affected patients (with lower physical functioning scales) reduces the mean of the data. This is shown in the study of Pendergrast et al. [18] where the housebound patients had a mean physical functioning scale of 17% while the non-housebound patients had a score of 42%. In the present study the mean physical functioning scale was 46%, with a range between 0 and 95%, indicating a large variation in ME/CSF severity. This strengthens the generalizability of the observed relations between VO_2_ and activity measurements and the physical function scale.

All three types of measurements in this study are related to the activity level of patients. Activity in women is partially determined by age, race, menopausal status, educational level, body mass index, depressive symptoms, smoking, chronic medical conditions, and pain [41]. The peak VO_2_ is influenced by genetics, gender, age, training status, exercise mode, bedrest, altitude, body composition, medication, the capacity of the respiratory and circulatory systems to take up and transport oxygen, and the capacity of the working muscles to receive and use oxygen. In ME/CFS patients the degree of fatigue/exhaustion, post-exertional malaise, underlying metabolic abnormalities, fibromyalgic pain, kinesiophobia and the use of medication may further influence physical activities.

A large number of studies have examined the validity of the SF-36 questionnaire, showing that the physical functioning scale discriminates between various diseases and healthy controls [42]. In ME/CFS patients Jason et al. reviewed the ability of the different subscales of the SF-36 questionnaire to discriminate CFS patients from healthy controls for the goal of operationalizing the measurement of “substantial reductions in previous levels of occupational, educational, social, or personal activities” [43]. The authors found that the physical functioning subscale was slightly less than optimal to discriminate between patients and healthy controls, using an area-under-the-curve (AUC) cut-off value of > 0.90 for optimal discrimination. In the community-based sample the AUC of the physical functioning scale was 0.84 and in the tertiary care sample 0.87. However, another study found an AUC for assessing substantial reductions of the physical functioning scale of 0.91 [44], suggesting that the use of the physical functioning score is valid with an acceptable sensitivity and specificity.

Several studies have shown a decreased peak VO_2_ in ME/CFS patients [45]. However, only one study determined the relation between the peak VO_2_ and accelerometer data in female ME/CFS patients: higher peak VO_2_ values were related to a higher physical activity time, physical activity energy expenditure, and a mean energy expenditure [46]. In the present study a significant linear relation was found between the peak VO_2_ and the number of steps as assessed by the Sensewear™ meter. Given the above mentioned large number of influencing factors on peak VO_2_ and physical activity in combination with the variation of steps on consecutive days, it is not surprising that, although a very significant relation between peak VO_2_ and the number of steps exists, the prediction intervals are wide.

There are also limited data available on the relation between peak VO_2_ and activity questionnaires. One study found a moderate association between exercise capacity and activity limitations/participation restrictions in patients with ME/CFS using the Chronic Fatigue Syndrome Activities and Participation Questionnaire [47]. Our data on the relation between peak VO_2_ and the physical function scale are consistent with their observations.

With respect to the relation between the physical functioning score and the activity meter, a previous study failed to demonstrate a difference in actometer-measured activity between two CFS groups, one with a mean physical functioning score of 54% and another with a mean score of 33% [48]. One case study noted a discrepancy between the psychometrically assessed improvement in function after graded exercise therapy versus the decrease in steps after therapy [49]. In the present study, we found a significant relation between the physical function scale and the number of steps; this was the strongest correlation in our study. The close correlation is not unexpected in ME/CFS patients as a previous study showed that ME/CFS patients are more aware of their daily physical activities compared to healthy controls [50]. Nevertheless, there is considerable variation between individual patients at specific levels of the physical functioning scale. For example, at the level of 30% of the physical functioning scale, the number of steps of individual patients varied between 1558 and 4266. At a physical functioning scale of 60%, the number of steps varied between 6277 and 9641, reflecting quite different function. The same holds true for the peak VO_2_ versus the number of steps taken. For a peak VO_2_ between 50 and 60% of normal the number of steps for individual patients ranged between 1135 and 4683.

Although the physical functioning scale is adequate to make a distinction between a group of diseased and non-diseased individuals, it is less useful to describe the degree of disability in individual patients, given the variation of the number of steps for a certain value of the physical functioning scale. An integrated approach of more than one type of measurement is therefore needed for the purposes of research study outcomes, disability determination, and individual patient management.

Our observation of the relation between the peak VO_2_, the number of steps, and the physical functioning scale is further strengthened by the observation that 20 patients who were re-evaluated because of worsening of symptoms had a significant reduction in peak VO_2_, the number of steps, and the physical functioning scale (Fig. 2). It is interesting to note that 1 of the 20 patients who presented with worsening of symptoms actually had an improvement in the number of steps/day and an unchanged physical function scale (individual data not shown). This confirms the importance of objective measurements that accompany symptom reporting.

It can be argued that the peak VO_2_ is the limit for a certain amount of activity: the lower the peak VO_2_ the less activity can performed or steps taken. Ideally, nomograms of the relation between peak VO_2_, number of steps per day, and the physical functioning scale should be available for ME/CFS patients, allowing clinicians to determine whether patients have gross over- or under-performance (in terms of number of steps) and explore the reasons for this over- and under-performance. Similarly, an imbalance between the physical function scale and the number of steps could be explored. This could also be beneficial for patient activity management. However, from the present data the construction of nomograms is not possible and future studies are needed.

Our data highlight a discrepancy between recommendations for CPET. In studies measuring %peak VO_2_, guidelines have advocated using the RER for assigning a test as maximal effort using an RER ≥ 1.1 [51]. In our subgroup analysis, 54% of the patients reached a peak RER ≥ 1.1 while 46% reached a peak RER < 1.1. Despite this difference (by definition P < 0.0001), the %peak VO_2_, the number of steps/day, and the physical functioning scale of the SF-36 were not different between patients reaching a peak RER ≥ 1.1 and patients with a RER < 1.1. Moreover, the relations between the three measurements: %peak VO_2_, number of steps per day, and the physical functioning scale were not different between patients with and without a RER ≥ 1.1. In ME/CFS patients a number of studies have shown that metabolic skeletal muscle abnormalities are present [5, 52, 53]. These skeletal muscle abnormalities may limit the maximal performance with RER values above 1.1. As Mezzani stated: “It must be borne in mind, however, that patients with severely impaired exercise tolerance can attain skeletal muscle strength exhaustion even earlier than central hemodynamic and ventilatory factors become limiting, interrupting exercise at peak respiratory exchange ratio values even lower than 1.00” [54]. Our data therefore suggest that an RER < 1.1 should not be used as an exclusion criterion for future studies of exercise performance in ME/CFS patients. Further support for this position comes from examining the fibromyalgia subgroup. In the present study 59% of the ME/CFS patients had a concomitant diagnosis of fibromyalgia. In all these patients, the reason for exercise termination was leg muscle pain in combination with leg muscle strength exhaustion. In these fibromyalgia patients the RER was significantly lower than in the ME/CFS patients without fibromyalgia. The low RER in fibromyalgia patients can be explained by the earlier exercise termination because of muscle pain. The %peak VO_2_, the number of steps and the physical functioning scale were not different between patients with and without fibromyalgia. This observation again argues against the use of a low RER as an exclusion criterion.

### Limitations

This was a retrospective analysis of data from patients with a maximum interval of 3 months between the three different measurements. We made the assumption that the clinical course would be stable over this period of time, but the retrospective nature of the study did not allow us to confirm this. A prospective study would be needed to evaluate the variability in measurements over time. We would emphasize that spontaneous rates of improvement in ME/CFS are approximately 3% over 18 months in some adult studies [55]. Given that patients were not offered new treatments during the assessment phase, we believe the likelihood of a major change in physical function was small.

Because of the limited number of patients, we did not correct for all the confounding factors as mentioned above for the three measurements. The Sensewear™ activity meter is not available anymore, but the present commercial actigraphs and smart watches have step measurements included. Only patients who were evaluated because of worsening of symptoms were re-analysed. We have no repeat data on patients who were stable or who improved.

## Conclusion

Disability grading or activity assessment in ME/CFS is most frequently performed using questionnaires like the physical functioning scale of the SF-36. Questionnaires can be augmented by adding more objective measures such as the number of steps on an activity meter and/or by adding peak VO_2_ data. The relation between the physical functioning scale and the number of steps, and the relation between activity (steps), perceived activity (physical functioning scale) and the maximum attainable activity (peak VO_2_) may give insight into a possible over- or under-estimation of the perceived activity and over- or under-performance of physical activities. Whether a better “energy management” leads to stabilization or improvement over time of patients needs to be studied. Finally, the presented standard deviations may aid the design of outcome studies.

## Data Availability

The datasets analysed in the current study are available from the corresponding author on reasonable request.

## Abbreviations

BMI: Body mass index
CFS: Chronic fatigue syndrome
CPET: Cardiopulmonary exercise test
ME: Myalgic encephalitis
VO_2_: Oxygen consumption
VCO_2_: Carbon dioxide release
%peak VO_2_: The VO_2_ at peak exercise normalized to a population reference value
